# Editorial: Innovative approaches to promote stroke recovery

**DOI:** 10.3389/fnins.2025.1657124

**Published:** 2025-08-06

**Authors:** Marianna Semprini

**Affiliations:** Istituto Italiano di Tecnologia, Genoa, Italy

**Keywords:** neurorehabilitation, non-invasive brain stimulation (NIBS), peripheral stimulation, motor control, rehabilitation assessment, digital technologies (DTs)

Stroke remains a leading global cause of disability and death (Feigin et al., 2022) and a substantial body of research is actively exploring various strategies to support its recovery. However, despite this extensive effort, only a limited number of findings are successfully translated into clinical practice. This gap is not solely due to the high cost of equipment or the need for specialized personnel, but also stems from a lack of robust clinical evidence demonstrating clear recovery outcomes. Additionally, the wide range of adjustable parameters within many interventions, makes it difficult to draw definitive conclusions about their effectiveness. Most studies also involve small, heterogeneous patient groups, which further limits the generalizability of their results.

In this Research Topic, we explore innovative approaches to enhance recovery following a stroke. We gathered a total of 18 manuscripts that together provide an overview of the most studied clinical challenges and proposed solutions in stroke rehabilitation. Half of these manuscripts are review articles focused on specific topics, allowing for an in-depth examination of various options, while the original research primarily involves exploratory strategies, with only two manuscripts reporting clinical trials.

The majority of studies concentrate on treatments for post-stroke symptoms, with only three addressing assessment methods. Broadly, the studies can be categorized into five groups (Figure 1, left): brain stimulation (six studies–5 of which are reviews), peripheral stimulation (3 studies–2 of which are reviews), motor control strategies (5 studies–1 of which is a review), patient-led rehabilitation (2 studies–1 of which is a review), and stratification and assessment methods (3 studies). Some studies span multiple categories.

**Figure 1:**
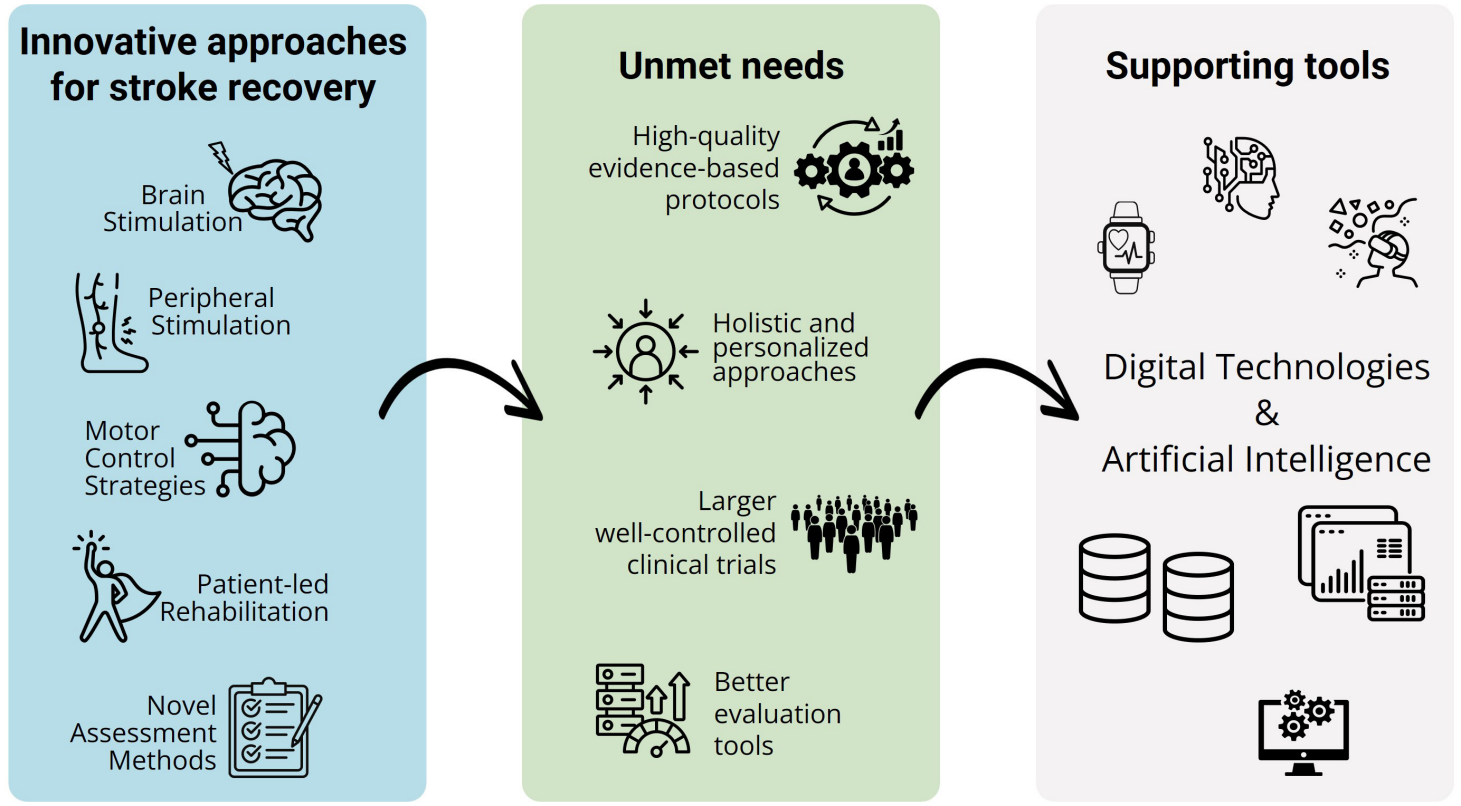
Innovative approaches for stroke recovery: most investigated areas (**left**), identified unmet needs (**central**) and tools to support future research (**right**).

Most research targets the chronic or sub-acute phases of stroke recovery, with only one study focusing on the acute phase within the first 24 h. Regarding symptoms, 10 manuscripts focus on motor impairments—two specifically on the lower limb and four on the upper limb. Additionally, two studies address central post-stroke pain, two focus on aphasia, one on dysphagia, and one on post-stroke fatigue.

While patient ethnicity is not reported in the manuscripts, it is likely that most studies involve populations from their respective regional contexts, suggesting a lack of diversity and limited information on other ethnic groups. This is an important factor, as technology-based solutions are typically developed and validated within specific demographic groups, and their effectiveness and translational potential may not readily extend to other populations (Bishop et al., 2025).

## Brain stimulation

Non-invasive brain stimulation (NIBS) techniques perform modulation of the central nervous system by electrically activating neurons in the brain and are used to influence cortical excitability, neuroplasticity, and behavior (Semprini et al., 2018). Their use in stroke rehabilitation has grown significantly over the past decades (Zhou et al., 2023; Veldema and Gharabaghi, 2022; Shen et al., 2022; Shah-Basak et al., 2023), and the studies included in this Research Topic add valuable insights to the existing body of knowledge in the field.

Liu et al. present a review on the effects of repetitive transcranial magnetic stimulation (rTMS) on central post-stroke pain (CPSP). By analyzing six randomized controlled trials (RCTs), the authors concluded that TMS can alleviate pain in CPSP patients and provide greater upper limb motor function improvement with respect to control groups, receiving either sham stimulation or conventional therapy. No significant effect of rTMS was found for treatment of cognitive symptoms such as depression and anxiety.

In their systematic review and meta-analysis, Gurdiel-Álvarez et al. also agree that rTMS could be considered a useful tool for CPSP treatment. However, they warn that there is low quality evidence for the effectiveness of rTMS on CPSP and that further and more rigorous studies are needed.

Wang C. et al. compared and analyzed the effects of different rTMS protocols on lower extremity motor function in stroke patients using network meta-analysis (NMA). They analyzed 38 studies and concluded that rTMS over the motor cortex benefits lower limb recovery using high frequency protocol for post-stroke time > 1 month and low frequency for longer post-stroke times. However, they advise further analysis and validation by high-quality RCTs to support their conclusion.

Wang Z. et al. explored the potential of cerebellar TMS for improving limb function after stroke. By reviewing clinical studies using this technique, they investigated its effectiveness, safety, and underlying mechanisms, highlighting advances in TMS and its combination with physiotherapy. The authors also examined the cerebellum's role in motor control, cognitive effects, and stimulation challenges, indicating that cerebellar TMS is a promising but complex tool for stroke rehabilitation, with recommendations for future research.

In their review, Yang et al. examined the potential of transcranial alternating current stimulation (tACS) as an alternative treatment for enhancing functional abilities in stroke patients. The studies reviewed indicated that tACS contributed to improvements in overall functional recovery, sensorimotor deficits, aphasia, and hemispatial neglect. Nonetheless, the exact mechanism through which tACS exerts its effects remains unclear.

Kwong Tang et al. propose a large double-blind randomized control trial in which transcranial direct stimulation (tDCS) will be assessed for treatment of post-stroke fatigue (PSF). Subjects will receive either active or sham stimulation over the motor cortex in two 20-min sessions per day for 5 days. A 4 weeks follow-up will evaluate change in fatigue severity using modified fatigue impact scale (MFIS). This study will demonstrate the benefits of tDCS in PSF treatment, paving the way for further research on optimal tDCS parameters.

Collectively, all these studies call for high-quality, evidence-based studies to support the potential of NIBS techniques in clinical applications to promote recovery from stroke.

## Peripheral stimulation

In addition to NIBS, other non-invasive techniques targeting stimulation of body periphery have recently emerged in the stroke field, with specific focus on promoting motor recovery.

In their review, Wang X. et al. investigate the effects of transcutaneous electrical acupoint stimulation (TEAS) for stroke rehabilitation. TEAS is a non-invasive technique that combines Chinese acupuncture and transcutaneous electrical nerve stimulation, which is delivered with low-frequency pulses to peripheral acupoints. By analyzing 16 trials, they found that indeed TEAS can promote upper limb function recovery. However, due to the limited number and low methodological quality of included trials, larger, high-quality multi-center studies are needed to confirm the results.

Hyeon Jeong et al. have developed an experimental protocol to examine the effects of combining peripheral nerve electrical stimulation (PES) with brain-computer interface-based action observation (BCI-AO) tasks on corticospinal plasticity after stroke, exploring how different PES pairings influence motor cortex activation. They found that task-driven corticospinal plasticity was higher when PES was applied synchronously with a highly attentive brain state during the action observation task, compared to continuous or asynchronous application. Although promising, their protocol only monitored corticospinal plasticity immediately after the task and did not assess retention. Further research is thus needed to evaluate the impact of this paradigm on long-term functional recovery after stroke.

Recently, transcutaneous vagal nerve stimulation (tVNS) has been used as a promising technique in neurorehabilitation context. Fan et al. have reviewed recent literature and confirmed that tVNS intervention is both effective and safe in treating stroke. However, the mechanism of action is still not fully understood and requires further exploration in the future.

Similar to brain stimulation research, peripheral stimulation techniques also need larger, well-controlled clinical trials to evaluate their effectiveness in stroke rehabilitation and how they might be integrated with standard therapies.

## Motor control strategies

Some studies leverage motor control theories and models to develop strategies that enhance movement recovery. For instance, brain-computer interfaces (BCIs) utilize neural activity to stimulate neuroplasticity (Shih et al., 2012). Within this Research Topic, there are two original studies focused on BCIs. One is the previously mentioned work by Hyeon Jeong et al., while the other was carried out by Sebastián-Romagosa et al., who examined a 25-sessions BCI treatment aimed at gait rehabilitation. This intervention proved effective in producing long-lasting improvements in gait speed among chronic stroke survivors. As a result, patients experienced increased lower limb movement, leading to improved and safer walking abilities, retained one-month post intervention.

Constraint-induced movement therapy (CIMT) has been employed for decades as an effective method to promote motor recovery by restricting the movement of the less-affected arm. CIMT improves upper extremity function by discouraging learned non-use and harnessing use-dependent neuroplasticity (Taub et al., 1999). Xu et al. reviewed CIMT research in the last 30 years and concluded that CIMT holds significant potential for further development in rehabilitation. Key focus areas include its combined use with other therapies, understanding its effects on motor cortex plasticity, optimizing intervention timing and dosage, and exploring new settings such as robot-assisted, telemedicine, and home-based rehabilitation.

An alternative approach involves suppressing abnormal motor activation to facilitate proper motor output. In their study Dewald et al. blocked undesirable and abnormal hand flexor contractions in persons post-stroke using local anesthesia of the median and ulnar nerves. Their findings indicate that many stroke survivors could experience better hand-opening when wrist and finger flexor activity was reduced through nerve block, particularly when functional electrical stimulation (FES) was applied to activate the typically weakened finger and wrist extensor muscles. This type of nerve block shows potentiality for stroke rehabilitation and could effectively overcome some of the limitations previously observed in FES treatments for stroke patients.

Recently, there has been growing interest in muscle strengthening, especially through eccentric training (ET), a well-established technique commonly used to enhance muscle strength in athletes, which involves contracting the muscle while it lengthens within the musculotendinous complex. Belghith et al. propose a novel comparison between ET and conventional therapy for improving outcomes in sub-acute stroke survivors. While preliminary evidence suggests ET can enhance muscle strength, stiffness, and walking performance, the specific biomechanical changes in paretic muscles remain unclear. This study will fill that gap, potentially guiding more effective early-stage stroke rehabilitation.

## Patients-led therapy

All manuscripts in this Research Topic focus on technological approaches as alternatives to standard therapy, but two of them stand out because they require patient-led actions.

Jiang et al. review the impact of mobile application-based interventions on post-stroke aphasia. They analyzed 15 studies, highlighting the potential of mobile app-based interventions to improve speech-language function in individuals with aphasia. However, more high-quality research is necessary to confirm their effectiveness across different areas and to explore the comparative benefits of various treatment methods.

Wei et al. describe a clinical study on 90 patients who received intravascular stent implantations immediately after ischemic stroke. They were interested in assessing the influence of a step-by-step inpatient rehabilitation program (SIRP) on the self-care capability and quality of life of patients. The observation group received SIRP in addition to routine nursing care, while the control group received only routine care. At admission, there were no significant differences between the groups. However, 3 months postoperatively, the observation group demonstrated significant improvements and also reduced complications and hospital stay duration. These results highlight the value of integrating structured rehabilitation programs into standard treatment procedures.

## Assessment methods

Throughout the rehabilitation intervention, the training program is continuously adjusted and monitored to optimize the patient's functional independence. This highlights the crucial role of assessment, emphasizing the need to go beyond traditional clinical scales (Garro et al., 2021).

Park and Kim analyzed 60 post-stroke individuals to determine whether conventional stratification strategies could improve the prediction of upper limb motor outcomes. They found that baseline upper limb motor impairment alone best predicted outcomes for less impaired or non-cortical subgroups, while combining it with brain structural damage improved predictions for others. Their conclusion is that applying stratification strategies, especially by initial impairment, enhances prediction accuracy beyond generic models, moving toward personalized prognoses for upper limb motor recovery after stroke.

Saab et al. present an original study focusing on predicting dysphagia treatment outcomes using speech recordings. The researchers developed a proof-of-concept model for automated dysphagia screening and tested its performance on training and validation cohorts. Their findings demonstrate that deep learning can effectively screen post-stroke dysphagia based solely on vocalizations. This approach paves the way for future non-invasive, objective, and rapid screening tools, potentially enhancing patient care, improving outcomes, and making swallowing assessments more accessible.

Wang et al. applied microstate analysis to compare EEG patterns between stroke patients and healthy controls, and examined correlations between microstate features and clinical scales in patients. They identified significant differences in resting-state EEG microstate features between stroke and healthy groups. Their findings suggest that EEG microstate analysis could offer valuable neurological insights for stroke rehabilitation and support its use as a potential neurological marker in clinical diagnosis and assessment.

## Perspective on current trends in stroke rehabilitation

This Research Topic provides valuable insight into current trends in stroke rehabilitation research and still unmet needs of current studies (Figure 1, central). While significant progress is being made in developing various treatment methods targeting different symptoms, there remains a notable lack of focus on assessment (Garro et al., 2021). This is a critical gap, as rehabilitation relies on a continuous cycle of assessment and treatment (Liu et al., 2022). Improved assessment tools are essential for accurately identifying patient needs, leading to more personalized therapies and ultimately better outcomes (Stinear et al., 2020). The rise of machine learning and digital technologies (Figure 1, left) presents a major opportunity to enhance assessment methods and make them more precise and effective, as indicated by the studies by Park and Kim, Saab et al., Wang et al..

Among treatment approaches, there is growing enthusiasm for non-invasive stimulation techniques—both central and peripheral—which aligns with the broader interest toward electroceuticals (García-Alías et al., 2020). BCIs and other plasticity promoting techniques are still being investigated and there is also a growing interest for patient-led rehabilitation strategies. However, there is a need for additional and larger studies in all these contexts.

Motor symptoms remain the most extensively studied aspect of stroke, yet stroke affects the brain as a network, leading to concurrent impairments in both motor and cognitive domains—the latter often being overlooked. In fact, cognitive aspects received only limited attention in this Research Topic.

Additionally, most studies tend to target individual symptoms rather than considering stroke location, cause, and other clinical factors. It remains to be addressed whether this approach overlooks critical factors and we advocate for future research to adopt holistic approaches incorporating comprehensive, objective assessments of patient function, supported by improved evaluation tools and personalized treatment strategies.

In this perspective, machine learning and digital tools present a valuable opportunity to develop more accurate and comprehensive models of stroke recovery (Silva and de Andrade, 2024; Gebreheat et al., 2024; Erol et al., 2020) (Figure 1, left). Importantly, future research should account for patients' ethnic and cultural diversity to accurately link health status to individual-specific factors (Ting et al., 2024). These elements may influence recovery outcomes and should be integrated into health models to enhance their relevance and effectiveness. Moreover, future studies should prioritize tailoring rehabilitation sessions to individual patient needs, exploring how to integrate various aspects of recovery.

In conclusion, this Research Topic provides a comprehensive overview of stroke rehabilitation at the intersection of laboratory investigation and clinical application. It not only highlights key areas of ongoing research but also outlines potential pathways to transition from the lab to clinical practice.
